# Features of the Psychoemotional status of Patients with Type 2 Diabetes Mellitus

**DOI:** 10.1192/j.eurpsy.2022.1007

**Published:** 2022-09-01

**Authors:** N. Chernus, L. Kamynina, R. Gorenkov, A. Sivkov, T. Savina, A. Serdakova, S. Sivkov, A. Zolotovickaja

**Affiliations:** The I.M. Sechenov First Moscow State Medical University: Moscow, Russia, The Outpatient Care Department, Moscow, Russian Federation

**Keywords:** anxiety-depressive, 2 Diabetes Mellitus

## Abstract

**Introduction:**

The study of the personal characteristics of patients with SD2 continues to be an urgent problem.

**Objectives:**

Study the features of the psychoemotional status of patients with type 2 diabetes mellitus

**Methods:**

The study included 62 patients with T2DM (HbA1c 7,3±1,3%) and visceral obesity (Grade 2) were included mean age 56.1 ± 2.4; BMI: 34.8 ± 2.3.. Research methods: the Beck test, the Hamilton scale, the Spielberger-Hanin questionnaire

**Results:**

Clinical signs of anxiety-depressive disorders were detected in (47) 75.8% of patients. - 1 group, 15 patients - 2 comparison group (without affective disorders). Beck’s test: 23.5 + 0.5 against 10.6 ± 0.3 and Hamilton’s shock 22.4 ± 0.4 against 7.9 + 0.2 revealed a reliable difference between groups (p < 0,001). In both groups of patients on the Spielberger-Hanin scale, moderate reactive anxiety was identified: 44.9 ± 0.7 versus 36.7 ± 0.5 point, which is significantly higher (p < 0,001) in patients of the 1 group. The level of personal anxiety 56.0 ± 0.5 versus 37.5 ± 0.4 points, which also revealed a reliable difference between groups (p < 0,001).

**Conclusions:**

The findings suggest a high incidence of anxiety-depressive disorders in patients of patients with T2DM and visceral obesity, which should be considered in pathogenetic therapy for these patients.

**Disclosure:**

No significant relationships.

